# A Scoping Review of the Utilization of Opioid Use Treatment, Harm Reduction, and Culturally Tailored Interventions Among Racial/Ethnic Minorities in the United States

**DOI:** 10.1007/s11469-024-01373-2

**Published:** 2024-09-16

**Authors:** Jerel M. Ezell, Elinor Simek, Netra Shetty, Mai T. Pho, Ricky N. Bluthenthal, Dawn A. Goddard-Eckrich, Sugy Choi

**Affiliations:** 1 Community Health Sciences, School of Public Health, University of California Berkeley, Berkeley, CA USA; 2 Berkeley Center for Cultural Humility, University of California Berkeley, Berkeley, CA USA; 3 Biology and Society, Cornell University, Ithaca, NY USA; 4 Department of Medicine, Section of Infectious Diseases and Global Health, University of Chicago Medicine, Chicago, IL USA; 5 Department of Population and Public Health Sciences, Keck School of Medicine, University of Southern California, Los Angeles, CA USA; 6 School of Social Work, Columbia University, New York, NY USA; 7 Department of Population Health, New York University, New York, NY USA

**Keywords:** Cultural tailoring, Harm reduction, Medications for opioid use disorder, Naloxone, Opioid use, Racial disparities

## Abstract

As part of a multilayered scoping review, we assessed literature on prevention and management interventions for racial/ethnic minorities in the United States (US) who non-medically use prescription opioids and/or who use illicit opioids such as heroin. The review specifically focused on access to and uptake of medications for opioid use disorder (MOUD) and harm reduction resources. We conducted a scoping review of peer-reviewed literature and governmental reports published between January 2000 and August 2024 on patterns of access to, and acceptability and utilization of, overdose prevention and opioid use management resources among racial/ethnic minorities in the US. Searches were conducted on Cochrane, PubMed, Embase, and Google Scholar, with us examining studies on the uptake of MOUD, such as buprenorphine and methadone, syringe services programs (SSPs), safe consumption sites, and harm reduction resources like naloxone (used to reverse overdoses) and fentanyl test strips (used to test for the presence of fentanyl in drug supplies). Additionally, we sought to identify and describe existing interventions for opioid use prevention and management that have expressly incorporated cultural adaptations related to racial/ethnic minorities’ specific needs and preferences in an effort to improve participants' sense of salience and acceptability and thus enhance utilization. We further endeavored to leverage this scoping review towards the development of research and intervention guidelines contoured to improve future scholarship and programming with these populations. The existing evidence suggests that racial/ethnic minorities in the US, specifically Black individuals, have diminished access to and/or utilization of preventive and management resources and amenities such as buprenorphine and naloxone, owing to structural deficits, provider bias, socioeconomic obstacles, geographic barriers, and communal stigma and distrust. Black individuals, relative to White individuals, also appear less likely to report using SSPs to obtain syringes and related resources, but across racial groups, those who used SSPs were more likely to be trained in, possess, and/or use naloxone. Further, there have been very few culturally tailored interventions for harm reduction or MOUD; there were limited data across the reviewed works on Native American/Indigenous or Asian populations; and the broader body of literature lacks methodological rigor. We close by proposing a cultural humility-focused model for better meeting the complex needs of these populations through research and primary and secondary intervention.

The contemporary opioid epidemic, fueled by heroin and fentanyl and non-medical use of opioid painkillers such as oxycodone and hydrocodone, has substantially impacted the well-being and functioning of communities across the United States (US). These impacts correspond to heightened morbidity and mortality rates, disruption of neighborhood cohesion, increased incarceration rates, and elevated healthcare and criminal justice costs (Das, 2023; Judd et al., 2023; Kuehn, 2021). Of particular note, the epidemic’s current so-called fourth wave has been characterized by the rising use of fentanyl and other synthetic opioids, and mixing of opioids *with* stimulants (e.g., cocaine and methamphetamines), contributing to steep spikes in fatal overdoses (Enos, 2024). The opioid epidemic’s greatest effects have increasingly been most concentrated, and felt most acutely, among racial/ethnic minorities, namely Black, Latino/x, and Native American/Indigenous populations in the country (Drake et al., 2020; Friedman & Hansen, 2022; James & Jordan, 2018; Valdez et al., 2022).

Harm reduction and medications for opioid use disorder (MOUD) represent “low-threshold” resources that can help curb opioid use-related risks such as overdose. MOUD represents a chemical treatment approach—focusing on aiding the normalization of brain chemistry and helping to relieve physiological cravings for opioids—that has proven potent in reducing opioid use-related morbidities (Mariolis et al., 2019; National Institute for Drug Abuse, 2021; Wakeman et al., 2020). 

There are three primary empirically supported MOUD modalities, including buprenorphine, methadone, and naltrexone. In this article, we focus on buprenorphine given its status as a standard of care that presents the lowest chance of overdose/iatrogenic effects (compared to methadone and naltrexone) (Bell et al., 2009). Buprenorphine can also be legally obtained from physician office visits and taken home, not requiring a patient to go to a specialty clinic, such as a methadone clinic (Wakhlu, 2009) which requires attendance and may have extensive wait-lists (Dunlop et al., 2017). Moreover, buprenorphine does not require a complete detox (compared to naltrexone) (Lee et al., 2018).

Access to Medicaid is important for making MOUD affordable, especially for lower-income racial/ethnic minority individuals. Although Medicaid coverage avenues have widened following the passage of the Affordable Care Act in 2010, some states rejected expansion, or impose restrictions, particularly states in the so-called Black Belt, a swath of Southern US states with large, Black populations, including Alabama, Georgia, Louisiana, and Mississippi (Lee et al., 2021). While Medicaid expansion has led to notable net decreases in uninsured rates, increases in Medicaid coverage across racial/ethnic groups, and reduced racial disparities in MOUD access, Black patients are still less likely to access MOUD (Johnson et al., 2022).

Racial/ethnic minorities may also be less likely to use MOUD and other harm reduction resources due to stigma and interweaving macrosocial barriers (Altekruse et al., 2020; Rosales et al., 2022). The implications of these disparities are increases in adverse health behaviors, such as injection equipment sharing (Allen et al., 2019; Bartholomew et al., 2020; Gibson et al., 2001), contraction of HIV and hepatitis C (HCV) (Koester et al., 2005; Naserirad & Beulaygue, 2020), and fatal overdose (Barboza & Angulski, 2020; Williams & Metzger, 2010). While our review does not directly address racial/ethnic differences in opioid use, several literature reviews have done so (Buonora et al., 2019; Chen et al., 2023; Ezell et al., 2024; Lee et al., 2019; Samuel et al., 2019).

On the harm reduction front, naloxone, an opioid antagonist that can be administered to help reverse overdoses (Miller et al., 2022), is considered a bedrock. The efficacy of naloxone is well-documented (Bachhuber et al., 2015; Barboza & Angulski, 2020). Crucially, in 2023, the Food and Drug Administration made naloxone available for over-the-counter purchase, but high costs are poised to disrupt uptake (Adams & Frost, 2023). To this end, public health departments often distribute naloxone kits for free, but patterns of awareness and access are mixed (Nolen et al., 2022). It is further important to note that some may hesitate to administer, or simply carry with them, naloxone due to fear of arrest or of administration worsening the overdose episode (Green et al., 2017). So-called Good Samaritan laws make it possible to report overdoses to authorities without fear of prosecution. To this end, these laws encourage overdose reporting (Moallef & Hayashi, 2021), making them especially important for racial/ethnic minorities implicated in and disproportionately criminalized through the so-called War on Drugs as users, dealers, or bystanders (Pamplin et al., 2023).

Syringe services programs (SSPs) are also increasingly important for providing naloxone and overdose prevention training and also in provisioning sterile injection equipment (Jones et al., 2021; Miller et al., 2022; Patel et al., 2023). Fentanyl, often mixed with heroin, is colorless and odorless, making it hard to detect (Green et al., 2020a, 2020b) and thus making exposure and risk reduction difficult to address. Fentanyl test strips (FTS) and other forms of "product" testing, reflecting more novel forms of harm reduction, allow one to test for various contaminants in drug supplies before drug use (Krieger et al., 2018) and are frequently offered in SSPs. However, SSPs are still broadly prohibited or “overpoliced” in the US. Relatedly, safe consumption sites (SCSs) can also provide a sterile environment for taking drugs in the presence of treatment staff but are comparatively nascent and not directly legally codified in the vast majority of the US (Altekruse et al., 2020; Khan et al., 2023; Rosales et al., 2022).

In considering barriers to MOUD and harm reduction utilization among racial/ethnic individuals, four general factors emerge: (1) limited awareness of the purpose and availability of such resources, (2) distrust of the quality and impact of these resources, (3) a lower concentration of such resources in racial/ethnic minority individuals’ communities, and (4) intracultural/internalized stigma against drug use and use of these resources.(Khan et al., 2023; Rosales et al., 2022).

Considering modifiable risk factors at the individual (client) level, Banks et al. (2023) illuminate the importance of culturally responsive interventions, operationalized as the process of identifying and registering a group's cultural norms and values, culturally "adapting" evidence-based interventions, and implementing novel strategies tailored to groups' cultural needs. Prior reviews in this space, including from Banks et al. (2023), have been limited in scope and not focused expressly on culturally responsive interventions for opioid use prevention or management for racial/ethnic minorities.

Scoping reviews can be leveraged to examine existing literature that has novel or ambiguous theoretical boundaries (Munn et al., 2018; Tricco et al., 2016), making this tool highly useful in contextualizing the presently amorphous contours of intervention strategies in racial/ethnic minority populations that use opioids. Along these lines, this scoping review has three goals. The first goal is to assess racial/ethnic minorities’ usage of MOUD and harm reduction resources, with an emphasis on patterns of access and client acceptability around these resources. The second goal is to highlight specific culturally tailored adaptations that have been developed as conduits for increased MOUD/harm reduction access and uptake. The third and final goal is to introduce conceptual practice and research guidelines for better characterizing and addressing the needs of racial/ethnic minorities at risk for opioid use-related harms through culturally attuned and responsive intervention.

## Methods

Between August 15, 2022, and August 21, 2024, we conducted a scoping review to identify and characterize the body of research on the racial and ethnic dimensions of MOUD treatment and harm reduction uptake in the US, with a focus on patterns of access, client acceptability, and cultural adaptations attempted for these interventions. This scoping review utilizes methods and approaches from Peters and colleagues (Peters et al., 2021). The data charting and synthesis were guided by the Preferred Reporting Items for Systematic Reviews and Meta-Analyses extension for Scoping Reviews (PRISMA-ScR) (Tricco et al., 2018). 

### Inclusion and Exclusion Criteria

An article or report was included in the scoping review if it met the following criteria: (1) it was published in a peer-reviewed journal or was a report from a government entity such as the Centers for Disease Control and Prevention; (2) it provided estimates or measurable details on access, acceptability or utilization patterns for opioid use treatment, harm reduction, or comparable opioid use prevention/management interventions among racial/ethnic minorities, namely, Black, Latino/a/x, Asian, and Native American/Indigenous individuals (i.e., differences in access to or utilization, according to one's race/ethnicity); (3) it contained data collected between at some point January 1, 2000, and August 21, 2024 (to provide a contextual trajectory into current patterns in the opioid epidemic, especially the current fourth wave); (4) the data were collected on individuals in the US; and (5) it was written in the English language. Articles were excluded if the study was conducted outside of the US or if there was no inclusion of non-White individuals. Moreover, we did not include conference abstracts. There were no restrictions on methods (e.g., quantitative, qualitative, etc.), study designs (e.g., cross-sectional vs. randomized), or sample sizes.

We located considerable variability in how treatments and harm reduction resources were described and how outcomes were measured. Additionally, we found substantial differences in the racial classifications that were used by authors. For example, some authors described their studies' participants as “Black” (sans ethnicity), while others noted both race and ethnicity of their samples (e.g., describing them as *non-Hispanic* Black), and in some cases, authors used race and race/ethnicity terms interchangeably. Likewise, in other instances, authors conflated ethnicity and nationality, as well as the term “Hispanic” (being from any predominantly Spanish-speaking country) and “Latino/a/x/ine” (being from a Latin American country, irrespective of primary language) (Martínez & Gonzalez, 2021). Such inconsistencies, among others later highlighted, precluded a meta-analysis and the computation of raw totals/percentages across the studies reviewed. Thus, we performed a scoping review of these elements, providing an overview, contextualization, and critique of existing literature, noting these limitations, as appropriate.

### Search Strategy

Articles were located and collated by two reviewers (JME and ES) using the keyword search parameters and permutations identified in the Appendix. A third person (SC) resolved disputes. To locate relevant articles, manual electronic searches were performed on the Cochrane Library to determine if a similar review existed (one did not), following this with structured, iterative searches on PubMed, Embase, and Google Scholar. Search keywords focused on programs and interventions addressing the elimination or reduction of harms associated with opioid/polydrug use (e.g., overdose, HIV/HCV contraction, harm reduction, naloxone) in racial/ethnic minority populations in the US. Briefly, search terms included common opioids (e.g., heroin, hydrocodone, codeine, fentanyl), specific administration routes (e.g., injection, snorting, smoking), racial and ethnic group classifiers (e.g., Black, African American, Hispanic, Latino/a/x/ine), and treatment/harm reduction resources (e.g., buprenorphine, methadone, naloxone, fentanyl test strips). The reference sections of collated articles were also reviewed for potentially relevant selections.

### Data Charting and Synthesis

PRISMA-ScR guidelines informed our charting and synthesis process (Tricco et al., 2018). Once the articles had been found, their references were loaded into Zotero, a citation management program. To ensure a degree of uniformity in charting approaches, both primary reviewers charted the same five articles and reached consensus on the subsequent abstraction techniques. The articles’ abstracts were read for relevant details and cues corresponding to article details were charted into a Microsoft Excel spreadsheet, using standard article trait parameters (How to Extract Study Data for Your Systematic Review, n.d.). Specifically, for articles that were provisionally deemed eligible, the following details were captured: first author name, year the article was published, year(s) the data were collected, the study design (cross-sectional, randomized, etc.), as well as resource/intervention type being discussed. During a second pass for the presence of racial/ethnic analysis, these additional details were captured: number of participants, the types of drugs used by the participants, and any stratification analyses done according to participants’ race/ethnicity. Details were kept regarding reasons for including or excluding articles in the final review.

Following the provisional scoping exercise, we then abstracted information from the articles corresponding to Rhodes’ (2002) four risk environment categories (*physical, social, economic*, and *policy*). We focused on the impact of structural impediments in order to contextualize the risk environments that best characterize the experience of racial/ethnic minorities who use drugs (Collins et al., 2019; McGowan et al., 2017). Key outcomes extracted from articles focused on rates of usage of a particular intervention or resource and assessed structural, meso and individual-level risk factors potentially corresponding to increased or decreased acceptability, access, and usage. We also initially considered differences according to age, income, education, and marital status, but these kinds of intra/intergroup differences were infrequently reported in the studies. Therefore, we removed these measures from consideration in the final scoping review, while acknowledging here their import in future analyses.

## Findings

**Fig. 1:**
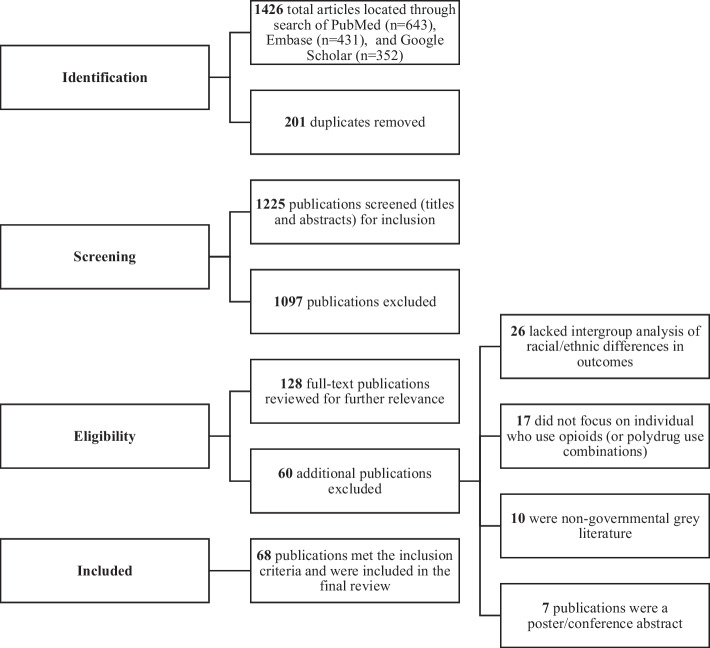
Scoping review flow diagram

Figure 1 outlines the article scoping review flow. Briefly, 1426 articles were located across PubMed (*n* = 643 ), Embase (*n* = 431 ), and Google Scholar (*n* = 352 ), from which 201 duplicates were removed. Of the 1225 articles that had their titles and abstracts assessed for relevance, 1097 were excluded for misalignment (i.e., they lacked connection to the research aims). This left 128 articles for full-text retrieval and further assessment. Of these, 60 were removed because they (1) lacked intergroup analysis of racial differences in outcomes (*n* = 26), (2) did not focus on individuals who use opioids (including polydrug use variations) (*n* = 17), (3) represented non-governmental grey literature (*n* = 10), or (4) represented a poster/conference abstract (*n* = 7 ). In total, 68 articles met the inclusion criteria and were included in the final scoping review.

### Use of MOUD, Harm Reduction Resources, and Culturally Tailored Adaptations Among Racial/Ethnic Minority Individuals

Below, we present findings from our scoping review, organizing our results into four sections: (1) Diminished MOUD Access and Treatment Uptake Patterns, (2) Disparities in Harm Reduction Services: Naloxone Access and Utilization, (3) Disparities in SSP and SCS Uptake and Product Testing Behaviors, and (4) Culturally Tailored Interventions for Racial and Ethnic Minorities. Selected studies are highlighted in Table 1.

**Table 1:** Overview of key articles included in the scoping review

Topical focus	Author(s)	Year published	Data collection period	Geographic setting	Key findings
General MOUD access/uptake					
MOUD	Buonora et al	2022	2018–2019	US	Among survey-takers with past-year opioid use disorder, factors associated with significantly lower odds of using buprenorphine without a doctor’s prescription included Black race, part-time employment, and no health insurance
MOUD	Handanagic et al	2021	2018	US	In a study of HIV-negative IDU, significantly fewer Black PWID (47%) used medication for opioid use disorder than Hispanics (65%) or White individuals (58%)
MOUD	Lagisetty et al	2019	2004–2015	US	Buprenorphine prescription was received at significantly more visits by White medical patients than patients of other races/ethnicities. Even after controlling for payment source, Black patients had statistically significantly lower odds of receiving a buprenorphine prescription at their visits
MOUD	Thompson et al	2024	2010–2019	US	In a medical prescription study, White patients with opioid use disorder were significantly more likely to be prescribed buprenorphine (*OR* 2.0; 95% *CI* 1.0–4.0)
MOUD	Wu et al	2016	2005–2013	US	In a study of people with OUD, controlling for receipt of public insurance, Black individuals and native-Hawaiians/Pacific-Islanders/Asian-Americans significantly underutilized opioid-specific treatment
MOUD access and quality					
MOUD	Barnett et al	2023	2016–2019	US	In the 180 days after the index event, patients received buprenorphine after 12.7% of events among Black patients, after 18.7% of those among Hispanic patients, and after 23.3% of those among White patients
MOUD	Dong et al	2023	2006–2020	US	In a prescription study, treatment duration differed significantly across racial and ethnic groups, with White patients consistently having the longest treatment durations. Racial and ethnic minority populations consistently had fewer buprenorphine episodes of at least 180 days vs White patients
MOUD	Dunphy et al	2022	2017–2019	US	Among Medicaid recipients, non-Hispanic Black, Indigenous, and Latino individuals had 42%, 12%, and 22% lower odds of buprenorphine receipt and 47%, 12%, and 20% lower odds of Vivitrol receipt, respectively, as compared to non-Hispanic White individuals
MOUD	Gibbons et al	2024	2013 and 2017	US	Medicare beneficiaries who were Asian/Pacific Islander, American Indian/Alaska Native, Black, Hispanic, and other/unknown race were 2.6 to 8.6% points less likely to receive buprenorphine or naltrexone than White beneficiaries
MOUD	Hadland et al	2017	2001–2014	US	Dispensing of a medication (buprenorphine or naltrexone) within 6 months of first receiving an OUD diagnosis was significantly less likely among non-Hispanic Black (105 [14.8%]) and Hispanic (1165 [20.0%]) youth compared with non-Hispanic White (17,119 [23.1%]) youth (*p* < .001)
MOUD	Hollander et al	2021	2015–2018	Allegheny County, PA	Of those receiving Medicaid through a supplemental security insurance program, Black enrollees were 18.2% less likely than White enrollees to start MOUD after controlling for gender, age, and Medicaid eligibility
MOUD	Kitsantas et al	2023	2010–2019	US	MOUD receipt was found to be higher among Black women, but their completion of treatment was lower compared to White women
MOUD	Landis et al	2022	2006–2014	US	Of Medicaid patients, non-Hispanic Black and Hispanic individuals had lower odds of receiving effective dosage (*aOR*s = 0.79 and 0.89, respectively) and sufficient duration (*aOR*s = 0.64 and 0.71, respectively), and lower odds of concurrent prescribing of opioid analgesics (*aOR*s = 0.86 and 0.85, respectively) and benzodiazepines (*aOR*s = 0.51 and 0.59, respectively)
MOUD	Manhapra et al	2015	2012	US	Black race (*OR* 0.39; 95% *CI* 0.35–0.43) was strongly and negatively associated with odds of receiving buprenorphine compared to methadone
MOUD	Miles et al	2023	2016–2019	US	Among Medicare disability beneficiaries, Black (adjusted hazard ratio [*aHR*] = 0.50; 95% *CI*, 0.47–0.54), Asian/Pacific Islander (*aHR* = 0.54; 95% *CI*, 0.41–0.72), Hispanic/Latinx (*aHR* = 0.81; 95% *CI*, 0.76–0.87), and other racial/ethnic groups (*aHR* = 0.75; 95% *CI*, 0.58–0.97) had a lower likelihood of timely buprenorphine than non-Hispanic White beneficiaries
MOUD	Xu et al	2023	2011–2016	US	Focusing on reproductive-age women with OUD, non-Hispanic Black enrollees in Medicaid were less likely to receive buprenorphine (adjusted odds ratio, *aOR* = 0.76 [0.68–0.84]) and more likely to be referred to methadone clinics (*aOR* = 1.78 [1.60–2.00]) compared to non-Hispanic White participants. Further, in adjusted analyses, non-Hispanic Black enrollees experienced greater discontinuation for buprenorphine and methadone than White individuals, but there was no difference in buprenorphine or methadone receipt or retention for Hispanic enrollees compared to White individuals
MOUD and geography					
MOUD	Amiri et al	2024	2015–2019	US	AI/AN had the longest median distances to opioid treatment programs (88 miles versus 4–10 miles) and buprenorphine providers (17 miles versus 1–3 miles) compared to other racial or ethnic majority block groups
MOUD	Dinardi et al	2022	2009–2019	US	An increased likelihood of exposure of White individuals to Black individuals in a county is associated with fewer substance use treatment facilities per 100,000, particularly those providing MOUD via buprenorphine and located in Northeastern and Midwestern counties. Also, a more unequal distribution of Hispanics is associated with fewer facilities per 100,000 providing MAT, and this association is strongest in Southern and Western counties
MOUD	Drake et al	2023	1999–2017	Continental US	Buprenorphine prescribers per 1000 residents within a 30-min drive decreased by 3.8 prescribers per 1000 residents in urban ZIP codes (95% confidence interval = − 4.9 to − 2.7) and 2.6 in rural ZIP codes (95% confidence interval = − 3.0 to − 2.2) whose populations consisted of ≥ 5% racial and ethnic minority groups
MOUD	Goedel et al	2020	2016	US	Counties with highly segregated African American and Hispanic/Latino communities had more facilities to provide methadone per capita, while counties with highly segregated White communities had more facilities to provide buprenorphine per capita
MOUD	Hansen et al	2013	2007	New York City, NY	Buprenorphine treatment rate was significantly (*p* < .01) negatively correlated with the percent of residents who are in poverty, Black non-Hispanic, and Hispanic. (ii) Methadone rates were significantly (*p* < .01) positively correlated with these variables
MOUD	Hansen et al	2016	2004–2013	New York City, NY	Buprenorphine treatment increased in all social areas over time with a significantly higher rate of increase in the social area with the highest income and the lowest percentage of Black, Hispanic, and low-income residents
MOUD	Hirchak et al	2022	2019	US	In metropolitan areas, Black people had a greater proportion of waivered buprenorphine prescribers than NHWs and Hispanic/Latinx adults. Availability of DEA-waivered prescribers was highest for predominantly AI/AN neighborhoods in Micropolitan, Small town, and rural areas, but lowest for predominantly AI/AN areas in metropolitan areas. Hispanic/Latinx adults experienced the lowest access to waivered providers overall
MOUD access during and after COVID-19					
MOUD	Nguyen et al	2022	2019–2021	US	After the onset of the COVID-19 pandemic, significant decreases in the estimated weekly rate of buprenorphine prescription fills per 1000 individuals (i.e., intercept) were observed for Black patients (2.5%; *p* = .009), Hispanic patients (4.0%; *p* = .009), and Asian patients (4.0%; *p* = .04) but not White patients (1.8%; *p* = .15)
MOUD	Wakeman et al	2022	2019–2021	Massachusetts	With a longitudinal focus, non-Hispanic Black patients had a lower likelihood of receiving buprenorphine compared to non-Hispanic White patients in the second period
Naloxone in medical settings					
Naloxone	Barnett et al	2023	2016–2019	US	In the 180 days after the index event, patients received naloxone after 14.4%, 20.7%, and 22.9%, respectively, and patients received benzodiazepines after 23.4%, 29.6%, and 37.1%, respectively
Naloxone	Kariisa et al	2022	2019–2020	25 states and DC	Rates of naloxone administration were lowest for Asian individuals (16.4%) relative to Hispanics (18.6%), White individuals (19.7%), Black individuals (20.3%), and Indigenous individuals (21.5%) among drug overdose decedents
Naloxone	Lane et al	2021	2018–2019	Cincinnati, OH	Of ED patients at high risk for OUD, non-White individuals had 62% lower odds of receiving naloxone than White individuals
Naloxone	Papp & Emerman	2023	2019–2021	US	Following an ED visit for opioid poisoning or overdose, White, Pacific Islander, and non-Hispanic patients were significantly less likely to receive naloxone prescriptions (*p* < 0.000)
Naloxone	Ray et al	2020	2011–2018	Indianapolis, IN	Overdose complaint and naloxone administration were more likely to occur among White than Black patients. White decedents were more likely than Black decedents to have had naloxone administered in the year before death
Naloxone	Reddy et al	2021	2017–2020	Providence, RI	No race-ethnicity inequities were observed in provision of take-home naloxone or referral to treatment after an ED visit for opioid overdose.
Naloxone	Weiner et al	2022	2018–2021	Massachusetts and New Hampshire	Hispanic/Latinx patients (visiting ED because of opioid-related overdose) were more likely to receive a prescription when compared to Non-Hispanic White patients
Naloxone in community-level and pharmacy contexts					
Naloxone	Chatterjee et al	2022	2014–2018	Massachusetts	Communities with greater percentages of people with Hispanic ethnicity were less likely to dispense any naloxone by naloxone standing order (aOR 0.91, 95% CI 0.86–0.96 per 5% increase)
Naloxone	Guadamuz et al	2019	2017	Philadelphia, PA	Naloxone was less likely to be available in pharmacies in predominately racial/ethnic minority neighborhoods (28.8%) compared with those with a large proportion of White residents (40.8%). Predominately racial/ethnic minority and low-income neighborhoods were less likely to offer naloxone without a prescription and less likely to be able to order it when compared with other neighborhoods
Naloxone	Green et al	2020	2013–2017	Rhode Island and Massachusetts	Factors associated with any naloxone doses dispensed and with a higher volume of naloxone dispensing at the pharmacy were communities where a larger proportion of the population was aged 25 to 44 years, had lower median household incomes, had a lower proportion population of White race, and more rural; neither race nor gender community characteristics was associated with naloxone dispensing outcomes in the multivariable analyses
Naloxone	Kinnard et al	2021	2017–2018	San Francisco, CA; Los Angeles, CA	Among PWID, White individuals were more likely to receive naloxone than Black individuals and Latinos in the past 6 months
Naloxone training and use					
Naloxone	Bennett et al	2022	2020	New York City, NY	During recent opioid use events, 64% reported never having naloxone and a person to administer present, which was more common among those who identified as non-Hispanic Black and those who experienced higher levels of stigma consciousness
Naloxone	Buresh et al	2020	2018	Baltimore, MD	There was a 9% rate of carrying naloxone among PWID and former-PWID with no significant difference between Black vs. non-Black individuals
Naloxone	Dayton et al	2020	2015–2020	Baltimore, MD	In a study of substance users in poor neighborhoods, White individuals had significantly higher odds of naloxone access, naloxone training, and naloxone use compared to Black individuals
Naloxone	Jones et al	2021	2016	Baltimore, MD	SSP clients, regardless of race, were more likely to have received overdose response training than Black non-clients. SSP clients and White non-clients were more likely to possess take-home naloxone than Black non-clients
Naloxone	Khan et al	2023	2019–2020	New York City, NY	Among illicit opioid users, naloxone coverage was greater in White (training 79%, possession 62%, daily access 33%, access during use 27%, and complete protection 13%, respectively) and Latinx (training 67%, possession 54%, daily access 22%, access during use 24%, and complete protection 16%, respectively) versus Black (training 59%, possession 48%, daily access 13%, access during use 12%, and complete protection 8%, respectively) participants
Naloxone	Kim et al	2021	2018	San Francisco, CA	Among PWID, receiving overdose response training was significantly lower among persons of non-White race/ethnicity compared to White individuals but higher among those who used SSPs
Naloxone	Kozak et al	2023	2020–2020	Baltimore, MD	In an urban methadone program, White race (*aOR* = 2.94, 95% *CI* 1.02–8.52) was associated with carrying naloxone
Naloxone	Nolen et al	2022	2016–2019	Massachusetts	In a public health study, non-Hispanic Black residents had a lower median number of naloxone kits for each opioid-related overdose death compared to non-Hispanic White residents
Naloxone	Oyler et al	2024	2020–2022	Eight counties in Kentucky	In a study of naloxone receipt and overdose-prevention education in behavioral health, criminal justice, and SSP programs, Black individuals were less likely than White individuals to receive naloxone and education in addiction-treatment and more likely to receive them in prison
Naloxone	Rodriguez et al	2024	2021–2022	Rhode Island	Of illicit-drug users surveyed, significantly fewer non-Hispanic Black individuals reported carrying naloxone compared to other races/ethnicities (*p* = 0.02)
SSP usage/enrollment					
Syringe Services Program	Burnett	2018	2015	20 US cities	In a study of HIV-negative IDU, 51% of Black individuals versus 53% of Hispanic and 54% of White individuals received syringes from an SSP in the prior 12 months
Syringe Services Program	Handanagic et al	2021	2018	US	In a study of HIV-negative IDU, fewer Black PWID received syringes from SSPs (40%) than did Hispanic (63%) or White PWID (63%)
Syringe Services Program	Iyengar et al.	2019	2016–2018	Miami, FL	A significantly greater percentage of PWID at the mobile van unit, compared to a fixed site, were Black/African-American (18.1% vs 4.9%, *p* < .001)
Syringe Services Program	Maurer et al	2016	1999–2014	Philadelphia, PA	The proportion of new registrants who identify as White has been decreasing, representing 51.5% of registrants in 1999 and 41.9% in 2014. Asians, Black individuals, and registrants identifying as other have not significantly changed over time. Latinos, the only group to have significantly increased, grew from 6.2% of new registrants in 1999 to 25.4% in 2014
Syringe Services Program	Parker et al	2021	2015, 2017	New York State	At the 10th and 90th percentile thresholds, Black individuals had higher odds of low syringe return ratios than White individuals (*aOR* = 3.03 and 1.86, respectively; *p*-values < 0.05)
Syringe Services Program	Pasman et al	2022	2019	Small miscellaneous community in Michigan	Among methadone-clinic patients, White race was associated with the likelihood of using an SSP, but observed no racial/ethnic differences in willingness to use SCS
Syringe Services Program	Rudolph et al	2010	2005–2007	New York City, NY	Among females (not PWID) in HIV-prevention programs, in contrast to IDUs using other syringe sources, those primarily using SSPs were less likely to be Black (adjusted odds ratio 0.26 [95% *CI* 0.11–0.57]
Syringe Services Program	Salow et al	2023		King County, Washington	Black, American Indian/Alaska Native, Latinx, and Native Hawaiian/Pacific Islander PWID were underrepresented as SSP clients as compared to White PWID
Syringe Services Program	Williams & Metzger	2010	2002–2006	Philadelphia, PA	Of PWID in HIV-prevention programs, Black individuals were less likely than White individuals to access syringes from SSPs (*OR* = 2.08; 95% *CI* = 1.48, 2.92)
SCS usage					
Safe Consumption Site	Pasman et al	2022	2019	Small miscellaneous community in Michigan	Among methadone-clinic patients, no racial/ethnic differences in willingness to use SCS were observed
Safe Consumption Site	Park et al	2019	2017	Baltimore, MD; Providence, RI; Boston, MA	Higher willingness to use a SCS was associated with racial/ethnic minority status among both injectors and non-injectors
Safe Consumption Site	Rodriguez et al	2024	2021–2022	Rhode Island	Of illicit-drug users surveyed, there were no significant racial/ethnic differences in the likelihood of using an overdose prevention center (i.e., a SCS)
Fentanyl test strip usage/preference					
Fentanyl test strips	Bailey et al	2023	2022	San Diego, CA and Tijuana, Mexico	Relative to White/non-Latinx people who inject drugs, those who were non-White/Latinx were significantly less likely to have used drug checking services (adjusted risk ratio (aRR) 0.22; 95% *CI* 0.10, 0.47)
Fentanyl test strips	Oh et al	2020	2018	San Francisco, CA	In a community sample of PWID, FTS use was lower among Black/African Americans as compared to White individuals (*aOR* 0.56, 95% *CI* 0.34–0.93)
Fentanyl test strips	Tilhou et al	2022	2021	Wisconsin	Among clients of SSPs, FTS use rates were significantly lower for non-Hispanic Black people than for non-Hispanic White or Hispanic people (*p* = 0.009)”

#### Diminished MOUD Access and Treatment Uptake Patterns

We located 26 studies assessing racial/ethnic differences in MOUD access and uptake, generally finding lower rates among Black individuals (relative to White individuals and Latinos). A lack of socioeconomic capacity, geographic access, resources, and healthcare infrastructure can be said to contribute most acutely to diminished MOUD uptake (Hansen et al., 2013, 2016; Kitsantas et al., 2023; Saloner & Cook, 2013; Wu et al., 2016). Here, we found that racial/ethnic minority individuals generally have substantially lower levels of access to and utilization of MOUD for non-medical opioid use, specifically buprenorphine (Barnett et al., 2023; Buonora et al., 2022; Drake et al., 2023; Dunphy et al., 2022; Gibbons et al., 2024; Hadland et al., 2017; Handanagic et al., 2021; Hollander et al., 2021; Lagisetty et al., 2019; Miles et al., 2023; Nguyen et al., 2022; Thompson et al., 2024; Wakeman et al., 2022; Xu et al., 2023), and less access to Medicaid as a likely facilitating factor (Beachler et al., 2021; Richard et al., 2020; Wood et al., 2019). Seven of the MOUD studies here addressed geographic considerations in buprenorphine settings. They highlight the existence of a two-tier system where buprenorphine prescribers are especially likely to be located in richer or whiter communities (Dinardi et al., 2022; Drake et al., 2023; Goedel et al., 2020; Hansen et al., 2013, 2016), whereas methadone clinics tend to be located in areas with relatively higher volumes and/or proportions of Black and Hispanic individuals (Goedel et al., 2020; Hansen et al., 2013, 2016). Studies also highlight a shortage of waivered buprenorphine providers in Indigenous metropolitan communities (Hirchak et al., 2022a, 2022b) and in Latino/x communities (Dinardi et al., 2022; Hirchak et al., 2022a, 2022b: especially in the South and West). Amiri et al. (2024) also reported that Indigenous individuals travel greater average distances for opioid treatment and buprenorphine providers relative to other races/ethnicities.

In a cross-sectional study of people who inject drugs who are HIV-negative, Black individuals’ use of MOUD (47%) was lower relative to Hispanics (65%) or White individuals (58%) (Handanagic et al., 2021). In an assessment of Medicaid claims dated 2017–2019, non-Hispanic Black, Indigenous, and Latino people had 42%, 12%, and 22% lower odds of buprenorphine receipt and 47%, 12%, and 20% lower odds of naltrexone receipt ( *p* < 0.001 for all), respectively, as compared to non-Hispanic White individuals, controlling for clinical and demographic patient variables (Dunphy et al., 2022). In an assessment of buprenorphine treatment using 2004–2014 data from the National Ambulatory Medical Care Survey and National Hospital Ambulatory Medical Care Survey, after accounting for payment method, sex, and age, Black, relative to White, patients had significantly lower odds of receiving a buprenorphine prescription during their visits (adjusted odds ratio (*aOR*), 0.23; 95% confidence interval (*CI*), 0.13–0.44) (Lagisetty et al., 2019). Xu et al. (2023) also found that, among reproductive-age women Medicaid enrollees, Black individuals were less likely than White individuals to receive buprenorphine, *aOR* = 0.76 [0.68–0.84], more likely to be referred to methadone clinics, *aOR* = 1.78 [1.60–2.00], and more likely to have both drugs discontinued, whereas there were no differences for Hispanic patients.

Cleavages during and after COVID-19 have also been witnessed. In a cross-sectional study of retail pharmacy claims for buprenorphine prescriptions during COVID-19, significant decreases in buprenorphine fills at COVID-19’s onset were observed only for non-White individuals (ranging from 2.5% for Black patients; *p* = 0.009 to 4.0% for Hispanic patients; *p* = 0.009) (Nguyen et al., 2022). Moreover, rates of buprenorphine fills decreased in level for (all) Medicare and cash-paying patients but decreased more for Black relative to White patients (Nguyen et al., 2022). In a cross-sectional study of buprenorphine treatment in Massachusetts during COVID-19, racial disparities emerged in the time period following COVID-19's onset, with non-Hispanic Black patients exhibiting reduced odds of receiving buprenorphine compared to non-Hispanic White patients in the second time period (*aPR*, 0.85; 95% *CI*, 0.72–0.99) (Wakeman et al., 2022).

Additionally, there may be disparities in MOUD treatment quality as concerns treatment administration, provider engagement, and patient retention. In a study of non-medical use of prescription opioids using data from the 2018 and 2019 National Survey on Drug Use and Health, the prevalence of self-reported use of prescribed buprenorphine was 6.8%, with Black race, relative to White race, being significantly associated with lower odds of using prescribed buprenorphine (*aOR*, 0.42; 95% *CI*, 0.18–0.98) (Buonora et al., 2022). In a study using Medicaid MAX data from 2006 to 2014, non-Hispanic Black and Hispanic patients had significantly fewer episodes of at least 180 days ( *p* < 0.01) (See also Dong et al., 2023), fewer episodes with an average daily dose of at least 8 mg ( *p* < 0.01), and were less likely to receive concurrent benzodiazepines and concurrent opioid analgesics ( *p* < 0.01) (Landis et al., 2022). In short, racial/ethnic minorities, especially Black individuals, tend to have less access to buprenorphine, may encounter segregation of methadone and buprenorphine services, and may experience disparities in treatment, even with MOUD that is obtained through Medicaid (Amiri et al., 2024; DiNardi et al., 2022; Goedel et al., 2020; Hansen et al., 2016; Hirchak et al., 2022a, 2022b; Hollander et al., 2021; Manhapra et al., 2020; Xu et al., 2023).

#### Disparities in Naloxone Access and Utilization

A total of 20 studies examined potential racial/ethnic disparities in naloxone access and utilization. Naloxone is vital to counteracting opioid overdose but is broadly underutilized in racial/ethnic minority populations (Barnett et al., 2023; Bennett et al., 2011; Buresh et al., 2020; Dayton et al., 2020; Khan et al., 2023; Kim et al., 2021; Kinnard et al., 2021; Kozak et al., 2023; Lane et al., 2021; Oyler et al., 2024; Rodriguez et al., 2024; Zinsli et al., 2023). For example, a multicity assessment of people who inject drugs in San Francisco, California and Los Angeles, California, from 2017 to 2018, found that Latinx (*aRR*, 0.53; 95% *CI* 0.39, 0.72) and Black (*aRR*, 0.73; 95% *CI* 0.57, 0.94) individuals, as compared to White individuals, were less likely to have received naloxone in the past six months (Kinnard et al., 2021).

The observed  curbing of naloxone usage is at least partly due to deficits in access/availability of naloxone in said communities: For example, in Massachusetts, communities with more Hispanics were less likely to dispense naloxone by standing order (Chatterjee et al., 2022). A study from the Massachusetts Department of Public Health also found that Hispanics (*aRR*, 0.78; 95% *CI*, 0.61, 0.99) and individuals of “other” race (*aRR*, 0.55; 95% *CI*, 0.40, 0.77) had lower rates of naloxone-kit receipt relative to White individuals (Nolen et al., 2022). In Philadelphia, naloxone was found to be more available in mostly White neighborhoods (40.8%) than in mostly racial/ethnic minority neighborhoods (28.8%), where pharmacies tended to require a prescription and less commonly ordered it (Guadamuz et al., 2019). However, Green et al., (2020a, 2020b) found no race differences in naloxone prescriptions in multivariate analyses.

Some studies on racial disparities in naloxone receipt came from medical settings, including hospital emergency departments. An analysis of emergency department patients at high risk for OUD in Cincinnati, Ohio, found that non-White patients had 62% lower odds of receiving naloxone than White patients (*aOR*, 0.38, 95% *CI* 0.14–0.99) (Lane et al., 2021). Barnett et al. (2023) also asked if people who had had non-fatal opioid overdoses received buprenorphine or naloxone within 180 days (in a variety of medical settings). There were significant racial differences in receipt both of naloxone (Black individuals, 14.4%; Hispanics, 20.7%; and White individuals, 22.9%) and buprenorphine (Black individuals, 12.7%; Hispanics, 18.7%; White individuals, 23.3%). However, in a study of 1348 unique patients in a Massachusetts healthcare system, Hispanic patients were more likely than White patients to receive a naloxone prescription because of opioid-related overdose (*aOR* 1.72, 95% *CI* 1.22–2.44) (Weiner et al., 2022). Another study found that following an ED visit for opioid poisoning or overdose, White and Pacific Islander patients were *least* likely to receive naloxone prescriptions (*p* < 0.000) (Papp & Emerman, 2023). In contrast, Kilaru et al. (2021) reported no differences between Black and White patients in naloxone claims following opioid-related ED encounters (*p* = 0.61).

Several studies pertained to people who died from overdoses. In a study of drug overdose decedents in 25 states and the District of Columbia, rates of naloxone administration were lowest among Asian individuals (16.4%) relative to Hispanic (18.6%), White (19.7%), Black (20.3%), and Indigenous individuals (21.5%) (*p-values not reported*) (Kariisa et al., 2022). In a comparable study done in Indianapolis, Indiana, White decedents, in contrast to Black decedents, were more likely to have had naloxone administered in the year before death (10.1% vs. 6.8%, *χ*^2^ = 4.0, *p* < 0.05, Cramer’s *V* = 0.05) (Ray et al., 2020). Noting race discrepancies in naloxone receipt, Reddy et al. (2021) introduced a medical intervention to reduce disparities. Their evaluation study found no statistically significant differences by race/ethnicity in the disbursement of take-home naloxone upon discharge (58.5% Black, 65.3% White, 71.2% Latinx, 83.3% “other” race; *p* = 0.087). However, of note, Black individuals were less likely to receive behavioral counseling.

Several studies also asked about racial/ethnic disparities in receiving training for naloxone administration, as well as possessing and using it. In Dayton et al.’s (2020) study in Baltimore, White, compared to Black individuals, had significantly higher odds of naloxone access (*aOR* 2.36; 95% *CI* 1.29–4.30), naloxone training (*aOR*, 1.93; 95% *CI* 1.11–3.37), and naloxone utilization (*aOR*, 2.02; 95% *CI* 1.17–3.50). Continuing, in a study of SSP and non-SSP clients in Baltimore, Maryland, Black SSP clients were almost four times as likely as Black non-clients to have received overdose response training (*aOR*, 3.85, 95% *CI* 1.88, 7.92). Moreover, White clients were nearly three times as likely to receive overdose response training than Black non-clients (*aOR*, 2.73, 95% *CI* 1.29, 5.75) (Jones et al., 2021). Black non-clients were also less likely to have take-home naloxone in their possession or at home (31%) compared to White non-clients (59%) or Black SSP clients (65%) and White SSP clients (50%) (*p* < 0.001). Kim et al. (2021) also found that Black people who inject drugs (PWID) were less likely than those of other races to report receiving overdose training; however, SSP clients were generally more likely to report receiving such training. A study of urban methadone patients showed that White patients were less likely than their Black counterparts to have naloxone ( *p* = 0.001) or to ever have received naloxone training (*p* = 0.048) (Kozak et al., 2023).

In short, neglect by providers in considering overdose prevention can lead racial/ethnic minorities to experience further morbidity/mortality risk. This can be compounded by the issues around decreased opioid treatment for pain.

#### SSP and SCS Uptake and Product Testing Behaviors and Attitudes

We located 18 studies on the racial/ethnic dimensions of utilization and acceptability of SSPs, SCSs, and product testing, considering if people do/will use the resource or service. In a study of IDU in New York City (Rudolph et al., 2013), Black individuals were less likely than White individuals to say SSPs were a primary source of syringes for them (*aOR* = 0.26 [*CI* 0.11–0.57]). Williams and Metzger (2010) not only noted similar findings in Philadelphia but also found Black SSP attendance had dropped during and after a nearby police intervention. In two national surveys of HIV-negative PWID, Burnett (2018) did not find significant race/ethnicity differences in reports of past-year receipt of syringes from an SSP (51% of Black individuals, 53% of Hispanics, 54% of White individuals) but the follow-up study (Handanagic et al., 2021) found less reported past-year receipt of syringes from SSPs among Black individuals (40%) than among White individuals (63%) or Hispanics (63%). Another study evaluated whether new client registration for SSPs in Philadelphia, Pennsylvania changed from 1999 to 2014. It found that proportions of new SSP registrants increased for Latinos (*F*(1, 14) = 113.08, *p* < 0.0001), decreased for White individuals (*F*(1, 14) = 6.43, *p* = 0.0237), and did not change for Asians (*F*(1, 14) = 3.57,* p* = 0.0796), Black individuals (*F*(1, 14) = 0.73, *p* = 0.4062), or those of “other” ethnicity (*F*(1, 14) = 0.20, *p* = 0.6584) (Maurer et al., 2016).

Another study in this broader space examined syringe return patterns in SSPs in New York, finding that Black individuals had higher odds of low return ratios than White individuals (*aOR* = 3.03 and 1.86, respectively; *p*-values < 0.05) (Parker et al., 2021). Additionally, a study looked at usage of fixed and mobile van sites providing syringes, determining that a greater percentage of PWID at the mobile van site were non-Hispanic Black/African American (18.1% vs 4.9%, *p* < 0.001) (no cross-racial analysis on overall SSP usage was provided)﻿ (Iyengar et al., 2019).

SSPs also frequently provide naloxone and training in how to use it. Several studies show that SSP clients, regardless of race, were especially likely to have received overdose training (Jones et al., 2021; Kim et al. 2021) and were more likely to possess take-home naloxone than Black non-clients were (Jones et al., 2021).

Owing to deep social stigma and attendant legal prohibitions, few SCSs currently operate in the US, so there was limited research on their usage/acceptability by race (or otherwise) (Park et al., 2019; Rodriguez et al., 2024). One recent study in Michigan with peer recovery coaches with lived experience found that those who identified as Black (*OR* = 0.361, *p* = 0.014) and other non-White individuals (*OR* = 0.338, *p* = 0.014) had lower odds of supporting SCSs, which, of note, are not formally authorized in the state at the time of this writing (Pasman et al., 2024). In a study of people who use opioids in Baltimore, Maryland; Providence, Rhode Island; and Boston, Massachusetts, researchers found that non-White drug users (Black, Latino, and “other race” combined) were significantly more likely than White individuals to be willing to use SCSs (*aOR* among injectors, 1.23; *p* = 0.01; *aOR* among non-injectors, 2.15, *p* < 0.001) (Park et al., 2019). At the time of this writing, the City of Providence has authorized the development of SCSs, while Baltimore and Boston have not, but both have legislation pending. Two other studies found no racial/ethnic differences in likelihood of using a SCS (Pasman et al., 2022; Rodriguez et al., 2024).

Other studies have examined and found racial disparities in use of FTSs. In a study of SSP clients in Wisconsin, FTS use rates were lower for non-Hispanic Black people than for non-Hispanic White or Hispanic people (*p* = 0.009) (Tilhou et al., 2022). Likewise, a study of people who inject drugs in San Francisco found that Black individuals had lower levels of FTS use relative to White individuals ( *p* < 0.001) (Oh et al., 2020). A study of people who inject drugs in San Diego, CA, and Tijuana, Mexico, found that those who were non-White/Latinx were significantly less likely to have used drug-checking services (Bailey et al., 2023).

#### Culturally Tailored Interventions for Opioid Treatment Among Racial and Ethnic Minority Individuals

Given the breadth of the structural factors implicated in the rise of opioid-related morbidities/mortalities in racial/ethnic populations, the need for cultural tailoring in client engagement, referral, and service delivery is a paramount consideration. Based on this review’s search criteria, we located only four studies with an explicit focus on cultural adaptations to opioid use prevention or management interventions, summarized in Table 2. As the studies demonstrated, culturally tailored clinical programs such as these contrast with “usual care” interventions that are typically formed around a White and Eurocentric standard in terms of foci on one's phenotypes and cultural sensibilities and preferences (Chin et al., 2007; Harvey & Afful, 2011; Kohn-Wood & Hooper, 2014).

**Table 2:** Overview of pilot culturally tailored interventions on opioid use prevention/management for racial and ethnic minority

Reference	Examples of approaches
Owczarzak et al. (2020)	“BMore Power” is a peer intervention model used by researchers to address drug-related hazards and harm reduction resistance in (at-risk) Black communities in Baltimore, Maryland • The program trained community members on safe drug use practices, naloxone administration, and provided education on other historically/politically relevant topics that pertained to drug usage (i.e., the War on Drugs) • Members noted their main obstacle during outreach was community distrust
Callejas et al. (2021)	“Promotes de Bienestar” (health promoters) is a New Mexico–based culturally tailored intervention, aimed at bringing awareness on OUD and approaches to naloxone administration to Latino communities • Focused on use of culturally relevant materials during interventions that bridged gaps in understanding of OUD (e.g., via infographics, English and Spanish text)
Hirchak et al. ( 2022b)	Development of a “Collaboration Board” intervention carried out with Indigenous communities in the Southwest • Emphasized idea that building a sense of belonging and connecting with one’s culture acts as a potential protective factor against OUD • Used the *Motivational Interviewing and the Community Reinforcement Approach*, focused on the culturally congruent use of “elders” and family (from their Indigenous communities), addressed cultural traditions and the importance of establishing trust between clients and providers, and targeted the stigmas associated with drug use
Zeledon et al. (2022)	Facilitation of a “focus group” to identify intervention components with Indigenous people in California Identified building a sense of belonging and connecting with one’s culture, including via drumming and sweat lodges, as potential protective factors to build upon in a culturally responsive intervention

Researchers devised a framework, Bmore POWER (Owczarzak et al., 2020), to address drug-related hazards and resistance to harm reduction in Black communities in Baltimore, Maryland. Using a peer intervention model, Bmore POWER employs local individuals with recent or current lived experience with drug use to conduct outreach in Baltimore neighborhoods with high levels of risk for drug use and overdose. Bmore POWER trainers, referred to as “members,” participated in a 10-part training addressing dynamics like safer injection practices, naloxone administration, etc., as well as the social and political touchpoints of drug use (e.g., War on Drugs), and strategies for community engagement. Trainers anecdotally described the intervention as empowering but cited deep community-level distrust and negative interactions with police as they performed outreach (Owczarzak et al., 2020).

Similar to this intervention, *Promotores de Bienestar* is a culturally tailored intervention focused on increasing awareness about OUD and building knowledge around the administration of naloxone in various Latino communities in New Mexico through the usage of (health) promoters, a type of community health educator or community health worker (Callejas et al., 2021). To develop the intervention, researchers held focus groups and related discussions with key stakeholders including Latino migrant workers and seasonal farmworkers and their children. Core components of the resultant intervention are related to the importance of using culturally resonant images and photos (i.e., containing Latinos), employing easy-to-read infographics, having English and Spanish translations, and emphasizing culturally empowered wellness (i.e., being strengths-based) instead of drug-related illness.

 Hirchak and colleagues describe convening a “Collaboration Board” to discuss methods of treatment for Indigenous populations in the Southwest with OUD, leading to the development of a strength-based and holistic implementation model focused on OUD recovery and general well-being (Hirchak et al., 2022a 2022b). Using the Motivational Interviewing and the Community Reinforcement Approach (MICRA) (Venner et al., 2021), the proposed intervention addressed intracultural stigma and focused on principles of motivational interviewing, family training, and building trust with providers and clients using culturally congruent Tribal members/ “elders.” The article also discussed macrosocial aspects of opioid misuse (e.g., historical and intergenerational trauma), of pain management, of opioids, and of Western and Indigenous medicine as potential factors associated with opioid misuse and recovery. Likewise, Zeledon et al. (2022) conducted focus groups with Indigenous peoples in California and identified building a sense of belonging and connecting with one’s culture as potential protective factors to build upon in a culturally responsive OUD intervention. As an example, one focus group participant, an individual in recovery, noted: “I think having our culture plays a major role in our recovery because it gives us something to turn to when we’re down and when we’re hopeless.”

To our knowledge, there is no published peer-reviewed research on a culturally tailored intervention for increasing racial/ethnic minorities’ knowledge or utilization of SSPs, SCSs, or product testing.

## Discussion

Across the studies reviewed, racial/ethnic minorities, relative to White individuals, were shown to consistently have lower levels of access to and/or utilization of MOUD (Dong et al., 2023; Hansen et al., 2013; Lagisetty et al., 2019) and harm reduction resources such as naloxone (Jones et al., 2021; Khan et al., 2023; Salow et al., 2023; Weiner et al., 2022). Limited access and use are driven by structural challenges including clinicians’ (implicit) biases, healthcare fragmentation, and geographic barriers as well as individual-level factors like limited health literacy and sociomedical distrust (which tie back to these broader structural challenges) (Eversman, 2015; Santoro & Santoro, 2018).

Even when receiving MOUD, minorities were less likely to be prescribed buprenorphine (in lieu of methadone) or to receive it for an adequate duration, including within drug treatment programs. Additionally, racial/ethnic minorities were generally less likely to receive naloxone even after overdosing. However, a study in an ED attempted to improve its medical overdose response and found no ethnic differences in medication administration but observed that Black individuals were less likely to receive counseling (Reddy et al., 2021). These findings highlight multiple consecutive/iterative barriers for racial/ethnic minorities in accessing opioid treatment, amplifying the odds of relapse and augmented or riskier forms of use. Even with health insurance, racial/ethnic minority patients may face barriers due to insurance issues (i.e., being uninsured or underinsured) (Mahajan et al., 2021).

During COVID-19, Medicare and self-pay patients, especially racial/ethnic minorities, had reduced access to buprenorphine, unlike Medicaid recipients. Individuals with Medicaid through Supplemental Security Income (SSI) were also less likely to initiate MOUD (Hollander et al., 2021), possibly due to stigma or concerns related to SSI or prior drug treatment (experiences). Second, there have been very few culturally tailored interventions developed to address these structural, meso, and individual-level deficits. For example, naloxone can be accessed through pharmacies, public health facilities, and SSPs, but disparities exist in its distribution and knowledge of acquisition methods. Black people may be less likely to carry naloxone or know how to obtain it, potentially due to fears of law enforcement/legal repercussions. Understanding both provider, community, and patient-level barriers is crucial for addressing disparities in naloxone access and use.

### Structural Challenges to Access/Use of MOUD and Harm Reduction Resources for Racial/Ethnic Minorities

Geographic access to buprenorphine-serving clinics varied by income and race/ethnicity, with wealthier and White clients having better access to buprenorphine providers and poorer and minority clients having better access to methadone clinics. Racial/ethnic minority communities tend to have far fewer MOUD options due to the general dearth of licensed providers in the nation’s historically disadvantaged and economically disinvested areas (“healthcare deserts”). Indigenous clients and Latinos in the West and Southwest face particular challenges in accessing buprenorphine care. This is mainly driven by healthcare fragmentation and socio-political resistance (i.e., providers’ and state/local lawmakers' concerns about the complexities of licensure and about MOUD “enabling” drug use) (Hudnall et al., 2022). These appear to be pronounced dilemmas in racial/ethnic minority communities (Bennett et al., 2011; Kinnard et al., 2021; Madden & Qeadan, 2020; Nolen et al., 2022).

Geographic disparities in naloxone access mirrored those of buprenorphine. Enhancing over-the-counter access to naloxone, an act currently being undertaken through pharmacies, could serve as an initial step in addressing these disparities, provided consumer purchase costs can be reduced. Other forces contributing to racial disparities in access/uptake, beyond geographic barriers, include social stigma against drug use, IDU, and MOUD, as well as misgivings about treatment purpose and efficacy, factors tied to health literacy and one’s negative, identity-related experiences with the healthcare system that may sow angst and deep institutional distrust (Ezell et al., 2020; Lister et al., 2020; Showers et al., 2021).

Crucially, at the root of these disparities are racist ideologies and policies that are generated and reproduced in communities through key institutions such as government, healthcare systems, educational institutions, housing entities, and financial institutions (Banaji et al., 2021). Therefore, primary interventions must first tackle these “upstream” structural and systemic determinants of disenfranchisement before population-level interventions are implemented and can be both fruitful and sustainable.

### Future Directions for Culturally Tailored Interventions and Research

Culturally tailored interventions that endeavor to eliminate or mitigate harms associated with opioid use among racial/ethnic minorities should focus on four dynamics (Table 3): (1) addressing policy and spatial deterrents to the mobilization of MOUD and harm reduction resources, (2) supporting client engagement and adherence around MOUD and harm reduction, (3) improving practitioners’ cultural humility and integrating culturally specific elements into interventions (Alvidrez et al., 2008; Davis et al., 2009; DeLoach et al., 2013; Dillard et al., 1992; Heron et al., 1997; Primm et al., 2002; Queener & Martin, 2001), and (4) measuring participants’ satisfaction with the cultural responsiveness of practitioners and interventions.

**Table 3:** Recommendations for developing culturally informed opioid use prevention and management interventions and research for racial/ethnic minorities

	Intervention	Research
Recommendation 1	Address policy and spatial deterrents to the mobilization of harm reduction resources, namely SSPs and SCSs, and MOUD in racial/ethnic minority communities and increase local capacity for training on and administration of naloxone and FTSs	To increase rigor and satisfaction with the research, utilize community-based participatory research methods to engage racial/ethnic minority clients in developing the research aims/scope and helping in data collection, interpretation, and dissemination of results (Wallerstein et al., 2017)
Recommendation 2	Interventions should focus on improving clients’ awareness, knowledge (e.g., on overdose risk factors, fentanyl test strip procedures, Good Samaritan laws), interest, capacity, and confidence/self-efficacy in relationship to addressing risk factors. Also, there should be a focus on mental illness and its connection to acculturative stress and microaggressions among racial/ethnic minorities, to better identify factors impacting access to/utilization of MOUD and harm reduction	To aid with general challenges in recruitment and retention of racial/ethnic minorities in research, utilize respondent-driven sampling (a representative method of recruitment/sampling that uses participants’ social network relationships (Heckathorn, 1997)) and venue-based sampling (e.g., recruiting at culturally salient places like cookouts, community fairs) to meet participants “where they are at”
Recommendation 3	Improve practitioners’ cultural humility by engaging in self-awareness exercises and focusing on integrating cultural amenities into interventions, such as culturally specific food, music, and art, and give the client a diary to capture how they feel about their experiences	When designing survey/qualitative instruments, note and be aware of the impact of the intricate social and cultural factors that contribute to racial/ethnic minorities’ opioid usage and harm reduction/MOUD engagement patterns, with a focus on intracultural stigma (Eversman, 2015)
Recommendation 4	Facilitate continuous evaluation, quality improvement, and enhance client satisfaction, practitioners should measure if clients believe they (the practitioner/organization) are culturally responsive, using tools such as the Cross-Cultural Counseling Inventory (Revised) (LaFromboise et al., 1991) or the Healthcare Provider Cultural Competence Instrument (Schwarz et al., 2015)	In considering the translatability of the research, be aware of how the study participants identify facilitators and barriers to better health and measure the “acceptability” or feasibility of the intervention to determine which interventions are most likely to be well-received and adhered to by the population

There are several ways to address policy and spatial deterrents to the mobilization of harm reduction resources, namely improving access to SSPs and SCSs, and MOUD in racial/ethnic minority communities. First, this process must include increasing local capacity for training and administration of naloxone and FTSs, including via active peer client engagement in training and service delivery. Addressing policy barriers to these interventions would include amplifying access to Medicaid (especially in states with existing partisan restrictions) to support MOUD implementation and developing richer and more culturally nuanced advertising and delivery of treatment and harm reduction resources  at SSPs, pharmacies, etc.

With policy and spatial barriers in mind, interventions with racial/ethnic minority clients should endeavor to support engagement and adherence to MOUD and harm reduction by targeting clients’ awareness, knowledge, interest, capacity, and confidence/self-efficacy in relation to addressing OUD risk factors through clinical engagements. To the matter of awareness, interventions should support clients in understanding the nature/causes of their opioid use, overdose risk factors/environments, and available mitigating resources. Knowledge-building should focus on imparting information on the social and biological causes of OUD and overdose, the purpose and value of mitigative resources, and technical/logistical instructions for access and usage (e.g., via training and demonstration). This could involve explaining Good Samaritan laws and how to use naloxone or FTS. A study conducted in New York City found that Black individuals, relative to White individuals, were less likely to have heard of Good Samaritan Laws (i.e., being able to report overdoses to authorities without fear of prosecution or other reprisals) (36.1% vs 60%) and were less likely to have accurate information regarding Good Samaritan Law protections (40.4% vs 49.6%) (Pamplin et al., 2023). This finding mirrors qualitative work in this space (Banks et al., 2021; Latimore & Bergstein, 2017; Seo et al., 2023; Winiker et al., 2020).

Interest-building should emphasize the collective cultural gain of community overdose/morbidity prevention efforts, to cultivate a sense of self-worth and intragroup connectivity (Ward & Brown, 2015). Capacity strengthening should focus on using policy to increase MOUD and harm reduction resources in racial/ethnic minorities’ communities. Lastly, interventions should seek to build self-efficacy, helping clients respond to the opportunities and barriers that come with accessing and adhering to MOUD and harm reduction. Along these lines, given the observation of an early-age drug use initiation window in certain racial/ethnic minority populations (Agyemang et al., 2022; Elliott & Jones, 2019), there is added value and potential in introducing these primary and secondary interventions during individuals’ adolescence.

Improvements in these five domains may also lead to deeper awareness of the factors underlying opioid use initiation/relapse and resistance towards adoption of MOUD and harm reduction. These underlying factors are relevant here because of mental illness’s acute connection to acculturative stress and microaggressions. Interventions should always bear in mind the outsize role of depression/anxiety, particularly in the context of racialized stressors, adjusting for and affirming the role that acculturative stress plays in the daily experiences of clients (e.g., via structural disenfranchisement in employment, healthcare, housing, law enforcement, and the judicial system and via recurring microaggressions) (Gómez, 2015; Smith et al., 2011; Zapolski et al., 2023).

Interventionists must understand that racial/ethnic minorities see, experience, and engage other people and the world in non-linear ways that are informed and grounded by their own cultural, spatially specific experiences. This awareness can come about by improving practitioners’ cultural humility and anti-racist orientation (actively opposing racism rather than solely not being racist). These paradigms both involve engaging in self-awareness exercises and integrating cultural amenities into interventions, such as intervention spaces with culturally specific food and music and/or decorated with artwork and posters of prominent racial/ethnic minority leaders, artists, and historical figures. Utilizing community-based partnerships focused on culturally responsive education can help to highlight and address the roots of intracultural stigma. A focus on culturally concordant care—integrating providers who share similar cultures and lived experiences into the clinical space—can also be uniquely impactful here (Hargons et al., 2022).

Adopting a strengths-based paradigm aligned with cultural humility (Brun & Rapp, 2001; Maton et al., 2004), interventions should also focus on working with participants to identify, openly discuss, and leverage the participants’ strengths. Participants can use a diary to outline their skills, goals, ambitions, and interests in relation to harm reduction and mental health (Coughlin & Smith, 2016; Jayadevappa et al., 2007) and to reflect on what is, and is not, “working” as they learn about and attempt to to access and consume MOUD and harm reduction resources. This can stimulate clients’ feelings of cultural identity, agency, and self-efficacy, help providers refine the intervention, and generate better rapport between racial/ethnic minorities and clinicians (and other community stakeholders, including emergency medical services (EMS) and law enforcement officials).

Providers and interventionists can measure the extent to which clients believe the practitioners are culturally responsive, using tools such as the *Cross-Cultural Counseling Inventory (Revised)* (LaFromboise et al., 1991) or the *Healthcare Provider Cultural Competence Instrument* (Schwarz et al., 2015).

With this in mind, culturally informed research for developing and piloting needs assessment measurements and interventions is greatly needed in this space. In particular, Community-Based Participatory Research (CBPR) is an inclusive, evidence-based approach that seeks to position research “subjects” as more than vessels for study, but as stewards of the research agenda who help with developing the research aims/scope, data collection, interpretation, and dissemination of results (Wallerstein et al., 2017).

In intervention research, it is particularly challenging to recruit racial/ethnic minority populations, especially those who use drugs (Venner et al., 2018). Respondent-driven sampling (RDS) (Heckathorn, 1997)—a method of recruitment that identifies and leverages participants’ social network relationships—is of particular utility for “hidden” populations such as drug users and racial/ethnic minorities (Daniulaityte et al., 2012; Duong et al., 2021; Rudolph et al., 2013; Wang et al., 2007). In RDS, the first participants enrolled, “seeds,” are asked to help recruit others they know who share similar traits/behaviors (such as race/ethnicity or drug use). RDS can be facilitated through a small number of initial seeds who are highly socially connected individuals from the target communities (as determined by, for example, community organization partners who know the target population well). When used with weighted sampling, RDS is a valuable methodological approach for generating a sample that is roughly representative of a specific group (Heckathorn, 1997; Salganik & Heckathorn, 2004). Recruitment can also leverage venue-based sampling (VBS), a method of “on the ground” community engagement wherein a research team creates a physical space or event (e.g., “block parties”) for interactions or otherwise recruits at culturally salient places (e.g., local fairs or galas) to facilitate connections between research staff and diverse communities (Fanzana & Srunv, 2001; Rao et al., 2017). Corresponding to CBPR goals, VBS lays the symbolic and material groundwork for meaningful community interactions (Ott et al., 2018). Given the institutional mistrust that exists for hard-to-reach communities (George et al., 2014; Tanner et al., 2015), it can be especially potent to meet racially/ethnically diverse communities *where they are at*. To help engender cultural congruence, recruitment should be aided by peers, people who formerly or currently use opioids, through events such as local barbeques/cookouts with music and popular local foods and fresh produce. The events should be coordinated and promoted in partnership with local stakeholders and a Community Action Board.

CBPR does not end at recruitment and sampling. Study participants should be involved in the formulation of research aims/goals, the curation of survey, and qualitative interview questions and also participate in data collection, manuscript preparation, and presentations (Israel et al., 2017). Awareness of the intricate social and cultural factors that contribute to racial/ethnic minorities’ opioid usage and harm reduction/MOUD engagement patterns is essential to the research process (Eversman, 2015) and this awareness most artfully manifests through the study participants themselves. In considering the translatability of the research, researchers must also be aware of how the study participants identify facilitators and barriers to better health and measure the “acceptability” or feasibility of the intervention, in light of logistic, financial, racial, and other socioeconomic factors, to determine which interventions are most likely to be positively viewed and adhered to by the population.

### Limitations

This review has some limitations. First, the study design of this review may have limited the articles included and hence the review’s findings. For example, because this study only included articles with measurable comparisons of racial/ethnic differences, it precluded studies with only one group or qualitative studies that did not incorporate quantitative assessments. Most studies included were exclusively quantitative in comparing treatment access or treatment quality. This likely led to few studies being included addressing stigma or lack of knowledge about resources, which are more often qualitative studies. The studies reviewed were also often conducted in cities (especially East Coast cities); therefore, the findings might be more relevant to those areas than to rural or semi-urban areas in the South, Midwest, or West.

There were also limitations created by the methodology of the reviewed studies. This included substantial variability in how MOUD treatment regimens and harm reduction resources were described by authors, specifically vis-à-vis the number of contacts with the client, volume of resource/service provided, etc. Furthermore, the reviewed studies did not consistently report the race *and* ethnicity of their participant samples; thus, it is possible, for example, that some individuals reported as “Hispanic” (ethnicity) were also Black (race), or vice versa. Asian and Native American people also tended to be highly underrepresented, if represented at all, in the studies included.

There were also limited studies on racial differences in usage of FTS, SSP, and SCS, thus preventing a more incisive assessment of disparities in these domains. Moreover, there was a dearth of interventions integrating cultural adaptations. To this end, there is a need to build greater synergies in how MOUD and harm reduction paradigms are developed, quantified, and categorized, with special attention to limitations imposed by culturally specific risk environments (Rhodes, 2002; Rhodes et al., 2003).

## Conclusions

Findings from this scoping review point to broad racial/ethnic disparities in multiple aspects of opioid use prevention and management, specifically among Black individuals. These disparities most centrally orbit around diminished access to and/or utilization of MOUD and naloxone, two key elements in the public health battle against the opioid epidemic (Ciccarone, 2021; Judd et al., 2023). Moreover, this review spotlights the greatly limited volume of cultural adaptations that have been fused into preventive and mitigative interventions. With this in mind, there is a stark need to develop, implement, and evaluate culturally adapted MOUD and harm reduction interventions to gauge other potentially effective means of support and health promotion of racial/ethnic minorities who use opioids.

## Appendix. Search terms utilized in the scoping review

Our scoping review utilized a broad array of search terms, and permuted terms, to better capture the wide spectrum of peer-reviewed articles potentially corresponding to the research aims. The search terms included the following: *abuse; addiction*, *buprenorphine*, *cocaine*, *crack*, *drug*, *drug use*, *fentanyl*, *fentanyl testing strips, harm reduction*, *heroin*, *injection*, *injection drug use*, *ketamine*, *LSD*, *methadone*, *methamphetamines*, *morphine*, *naloxone*, *naltrexone*, *Narcan*, *needles*, *needle exchange*, *needle exchange program*, *non-medical drug use*, *opioids*, *opioid use*, *opioid use disorder*, *overdose*, *overdose prevention center*, *painkillers*, *polydrug use*, *prescription*, *product testing*, *safe consumption sites*, *stimulants*, *synthetic*, *syringes*, *syringe exchange program*, *syringe services program*, *substance use*, *substance use disorder*, and *suboxone.*

Permuted terms also incorporated signifiers of racial/ethnic identities, including the following racial/ethnic categories: *African American*, *American Indian*, *Alaskan*, *Asian*, *Asian American*, *Black*, *Caucasian*, *Hawaiian*, *Hispanic*, *Indian*, *Indigenous*, *Latino/a/x/ine*, *Native American*, *Pacific Islander*, and *White.* We further utilized specific nationalities for Hispanic groups that are prevalent in the US, using the following descriptors: *Cuban*, *Dominican*, *Mexican*, and *Puerto Rican*. In this review, we use the expression “Indigenous” to reference Alaskan Natives, American Indians, and Native Americans. We further use the term “Asian” to refer to Asian Americans and Pacific Islanders.

## Abbreviations

aOR: Adjusted odds ratio
FTS: Fentanyl test strips
HCV: Hepatitis C
IDU: Injection drug use
MOUD: Medications for opioid use disorder
OUD: Opioid use disorder
SCS: Safe consumption site
SSP: Syringe services program

## Declarations

No human subjects were included in this study.
